# Media coverage of violence against women in India: a systematic study of a high profile rape case

**DOI:** 10.1186/s12905-015-0161-x

**Published:** 2015-01-22

**Authors:** Mark Phillips, Fargol Mostofian, Rajeev Jetly, Nazar Puthukudy, Kim Madden, Mohit Bhandari

**Affiliations:** Department of Life Science, McMaster University, Hamilton, Canada; Department of Clinical Epidemiology & Biostatistics, McMaster University, 293 Wellington St. N, Suite 110, Hamilton, ON L8L 8E7 Canada; Division of Orthopaedic Surgery, McMaster University, Hamilton, Canada

**Keywords:** Delhi, Gang-rape, Media coverage

## Abstract

**Background:**

On December 16, 2012 a 23 year old female was gang-raped on a bus in Delhi. We systematically reviewed professional online media sources used to inform the timing, breadth of coverage, opinions and consistency in the depiction of events surrounding the gang-rape.

**Methods:**

We searched two news databases (LexisNexis Academic and Factivia) and individual newspapers for English-language published media reports covering the gang-rape. Two reviewers screened the media reports and extracted data regarding the time, location and content of each report. Results were summarized qualitatively.

**Results:**

We identified 534 published media reports. Of these, 351 met our eligibility criteria. Based on a time chart, the total number of reports published increased steadily through December, but plateaued to a steady rate of articles per day by the first week of January. Content analysis revealed significant discrepancies between various media reports. From the 57 articles which discussed opinions about the victim, 56% applauded her bravery, 40% discussed outrage over the events and 11% discussed cases of victim-blaming.

**Conclusions:**

The global media response of the December 16th gang-rape in India resulted in highly inconsistent depiction of the events. These findings suggest that although the spread of information through media is fast, it has major limitations.

## Background

On December 16th, 2012 a 23-year-old female student was the victim of a gang-rape on a moving bus in Munirka, South Delhi. The victim and her boyfriend had boarded the bus around 9:15 PM, where the six men on the bus, including the driver, attacked them. The boyfriend was physically assaulted while the female student was gang-raped (i.e. the female was raped by several males) before being thrown out of the moving bus approximately 45 minutes later in Mahipalpur- a neighborhood in New Delhi [1]. The accused then attempted to run over the woman and her boyfriend as they fled in the bus [2]. The woman and her boyfriend were rushed to the All India Institute of Medical Sciences (AIIMS) before the female victim was transferred to Safdarjung Hospital in South Delhi. Doctors commented that the victim suffered from serious injuries to her face and head, and was in serious condition [3]. On December 19th, the woman had undergone her fifth surgery to remove damaged intestine. She was still in critical condition, yet stable [4]. On December 26th, 2012, the woman was transferred from Safdarjung Hospital via aircraft [5] to Mount Elizabeth Hospital in Singapore, where her health status was deemed to be deteriorating due to severe organ failure. Her major injuries included lung and abdomen infection as well as brain injury [6]. The woman eventually succumbed to her injuries and died of multiple organ failure on December 29th, 2012 [7]. The woman’s body was brought back to Delhi later that day via chartered airplane [8]. She was cremated almost immediately after her return to India on the morning of December 30th in order to avoid a large media presence at the event [9]. The news of this event spread throughout the world, resulting in protests against the current treatment of women and laws against rape in India [10].

Protests and demonstrations were held to pay respects to the victim and bring attention to the mistreatment, rape and degradation of women in India. The protests turned violent in India, and resulted in the death of a police officer who was working to control a crowd of protestors in Delhi. There was controversy over the cause of the officer’s death, although witnesses have stated that he was attacked and beaten by a small group of protesters [11]. The demonstrations spread globally and included a protest in Toronto, Canada [12] and Melbourne, Australia [13].

Media is a tool of mass information spread to the global community when an event such as this occurs [14]. Media professionals must understand their ability to aid in the shaping and development of an appropriate response to situations within health-related fields [15]. The information of this particular rape case in South Delhi was spread world wide in a matter of days through the professional media. Previous research that analyzes the dissemination of information throughout professional media has not studied instances of violence against women as the event that triggers the media spread. Social movements are capable of providing transformation of current cultural aspects, particularly when a crisis arises that results in a rapid uprising. Media’s presentation of controversial events provides numerous tools in which individuals are drawn to the social movement, making media a major tool in the development of social movements [16]. It is important to understand how the spread of media information affects social movements after a rape event within the local and global population, as previous reports suggest that media plays a crucial role in social movements and the formation of global protests [17]. A thorough understanding of media’s role in the spread of information regarding rape and other women’s health issues as a spark for social movements is valuable in aiding in current and future women’s health movements.

The objectives of this systematic review of professional media sources are to inform and visually represent media’s role in spread of the information from a local to a global scale, using the December 16, 2012 gang-rape in Delhi as a case example, and outline the capabilities that media possess to spark social movements regarding policies to protect women. The report also provides a content analysis of information distributed by the media regarding this specific rape case.

## Methods

This study applies the methods of a systematic review to examine media coverage of the gang-rape in Delhi.

### Data source

We used two comprehensive databases (LexisNexis Academic and Factivia) to conduct this media analysis. These databases provided large online collection of sources and included a comprehensive search function similar to journal databases like Pubmed. LexisNexis search functions includes over 350 full newspapers, legal proceedings and company information (http://www.lexisnexis.com/hottopics/lnacademic/). Factivia database provides access to newspaper, television and radio transcripts, web and blog content, profile for companies and photographs. (http://new.dowjones.com/products/factiva/) We adapted previously published methodology in our search strategy [15].

### Search strategy

Two reviewers piloted a preliminary electronic search using a variety of relevant search terms, and selected 5 media reports that were appropriate to the topic. Key terms were identified from these media reports and used to develop our main search strategy (Table 1).

**Table 1:** **Search strategy**

**Search sources/databases**		**Search terms**	**Search strategy**
Local Delhi	Television	1. 23 year old	1 and 2 and 7
○Business Standard	○CNN	2. Bus	1 and 2 and 8 and 10
○Hindustan Times	○NDTV India	3. December	1 and 6 and 7
○India Today	○CBC	4. December 16	2 and 4 and 7
○Tehelka	○BBC	5. December 2012	2 and 6 and 10
○Zee News	○CTV	6. Delhi	2 and 6 and 7
Indian National	○CityTV	7. Gang rape	4 and 6 and 10
○The Times of India	○Global	8. India	4 and 6 and 7
○The Hindu	Europe	9. Medical Student	4 and 9 and 7
Canadian National	○BBC World News	10. Rape	5 and 6 and 10
○Globe and Mail	Other/Social Media	11. South Delhi	5 and 6 and 7
○The National Post	○Blogs by professional newspapers	12. Student	6 and 10 and 12
○Huffington Post	Newspaper Databases		6 and 7
United States	○LexisNexis Academic		7 and 11
○Washington Post	○Factiva		
○New York Times			
○Wall Street Journal			
○USA Today			

We searched the electronic newspaper databases LexisNexis Academic and Factiva for relevant media reports that were published from December 6, 2012 to January 7, 2013, using a combination of the identified search terms (Table 1). Additionally, we conducted a manual search of several newspaper and television websites using the same search terms. One reviewer conducted the systematic search of media reports.

### Inclusion and exclusion criteria

Newspaper articles, online television videos, webcast, blogs and other forms of professional media were included if they fulfilled at least one of the following criteria: 1) description of the events, 2) rape victim’s condition, 3) rape victim’s death, 4) protests as a consequence of the event, 5) testimonies of events from victim, witnesses or the accused. Additionally all media reports included were in the English language published from December 16, 2012 to January 7, 2013. Professional media is defined as any source of news produced by trained journalist and accredited to a licensed broadcaster. Only English sources were chosen because of the authors’ inability to interpret or translate sources in other language sources. English is a very commonly used language for international articles, which is why we felt that the inclusion of only English articles would be sufficient.

Media reports were excluded for one or more of the following reasons: 1) Focus not on the specific gang-rape in New Delhi, 2) Focus on police conduct 3) Focus on the trial/ court case, 4) Focus on government response to the events, 5) Focus on suicide of the accused, 6) Social media sources authored by non-professional sources 7) Focus only on emotional response or opinion of family and friends of victim. We refrained from inclusion of police conduct and information regarding the trial and court case because it was not directly related to the events of the gang-rape and introduced dimensions of what is considered criminal justice, which was beyond the scope of this study. The trials began on January 7^th^, 2013. This is also the reason for our timeline ending on the aforementioned date.

The focus of this study was on published professional media, thus social media sources and any media reports not authored by professional journalists were excluded. Additionally, we identified that most articles after January 7, 2013 did not focus on the events of the December 16, 2012 event, thus all we limited our search to date range from December 16, 2012 to January 7, 2013.

Two reviewers independently screened the media reports for inclusion based on the title and full-text using the above eligibility criteria. We resolved all disagreements by a consensus process that required the reviewers to discuss their rationale for their decision. If consensus could not be reached a third reviewer (another author) was consulted. In the case of exact duplicate media reports with differing dates of publication, the earlier publication was included, due to the time sensitive nature of this research. Additionally, if a duplicate report appeared but from different sources both media reports were included.

### Data collection and data synthesis

Reviewers abstracted information on each media report using an *a piori* data abstraction form created by one of the authors of the study. The forms included the following headings: the date and time of publication, the media source and its location focus, which included regional (i.e. Delhi), national (i.e. India), or continental (i.e. Indo-Asia) coverage in some cases.

All included media reports were then assessed by the reviewers and grouped based on location, date (converted to India Standard Time (IST) for consistency) and theme. The theme categories were determined a priori, and included: 1) Description of event, 2) Victim’s health condition/medical decisions, 3) Protests as a result of the event, 4) Testimonies (victim, victim’s friends, witnesses, accused or family member), 5) General public’s response to the event (excluding protests).

These grouping categories were then used to analyze the media reports for content and their sources of evidence. The reports in each theme category were compared for similar content, representation of events and sources of evidence use to support claims. The results were then reported using descriptive statistics (frequency and percent of reports which shared these characteristics).

Additionally, a visual map of the transfer of information from December 16, 2012 to January 7, 2013 was created. This map is based on an online tool called the “Harassmap” created in Egypt to track cases of sexual harassment [18]. Number of media reports from each location, the date and time of first publication for each location category were represented on a world map, to show transfer of information from local Delhi to international news sources. For news reports that did not report time of publication, attempts were made to contact editors and authors. If no time of publication was determined, papers were reported based solely on the date. Additionally a histogram of the number of news reports daily from December 17th, 2012 to January 7th, 2013 was created. The cumulative number of reports since December 17th, 2012 was also plotted.

### Assessment of agreement

We conducted inter-rater agreement for title/full text screening step using a weighted kappa (κ) statistic. We decided *a priori* that Cohen’s κ values of less than 0 were rated as less than chance agreement; 0.01-0.20, slight agreement; 0.21-0.40, fair agreement; 0.41-0.60, moderate agreement; 0.61-0.80, substantial agreement; and >0.80, high agreement [19]. All agreement analyses were conducted using SPSS v.18.0 (IBM Corp., Armonk, NY, USA).

## Results

### Search results

**Figure 1:**
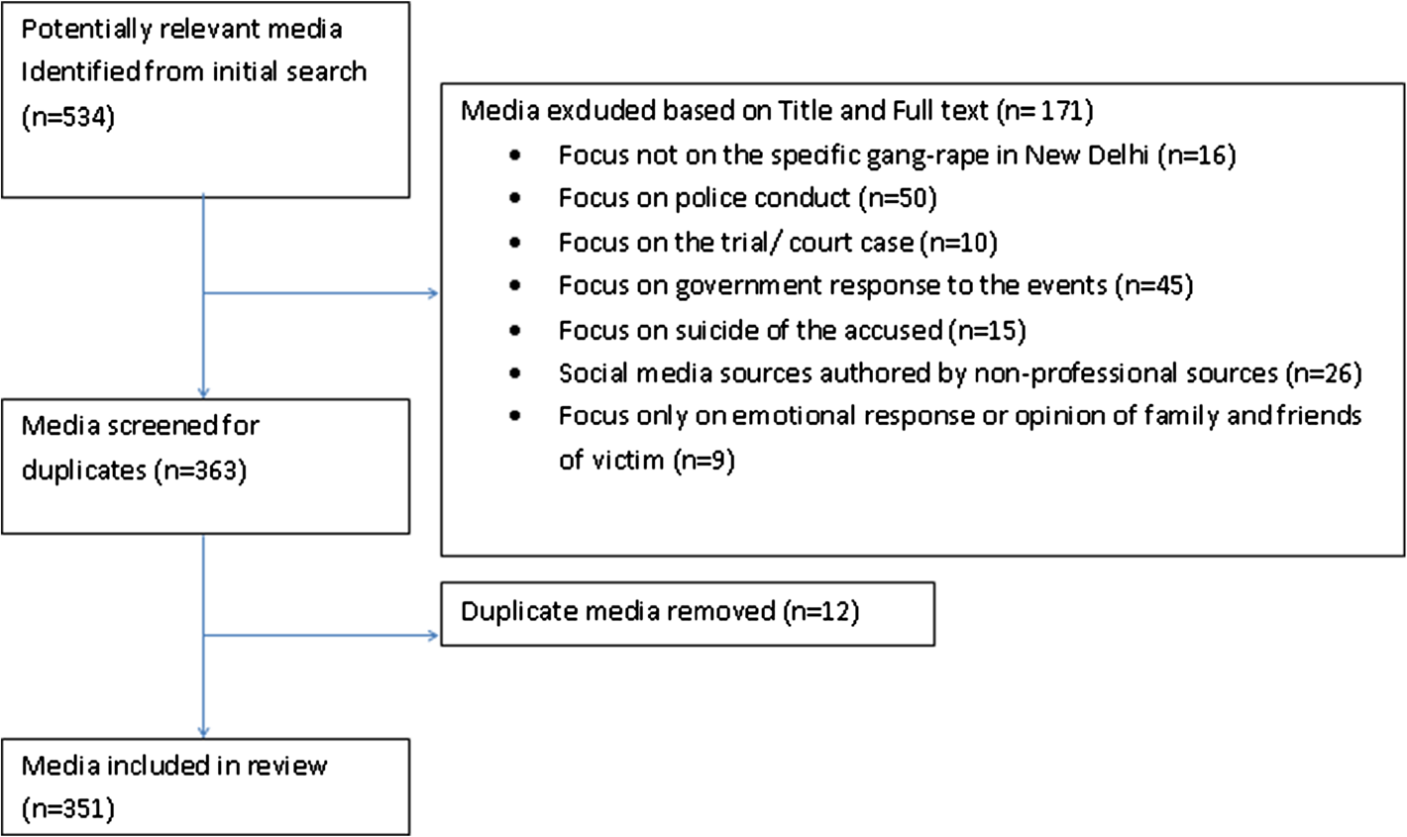
**Screening flow diagram.**

Our media search identified 534 potentially relevant citations and 351 total media reports were relevant for inclusion (Figure 1). 171 media reports did not meet our inclusion criteria and 12 media reports were duplicates. Inter-rater agreement was fair for the title and full text screening stage (κ = 0.50, 95% Confidence Interval (CI): 0.436-0.558).

### Characteristics of reports

From the 351 included media reports; 26 were categorized as description of event, 112 as description of victim’s health conditions or medical decisions, 134 as description of protests as a result of the event, 24 as testimonies (victim, victim’s friends, witnesses, accused or family member), 55 as general public’s response to the event (excluding protests).

**Figure 2:**
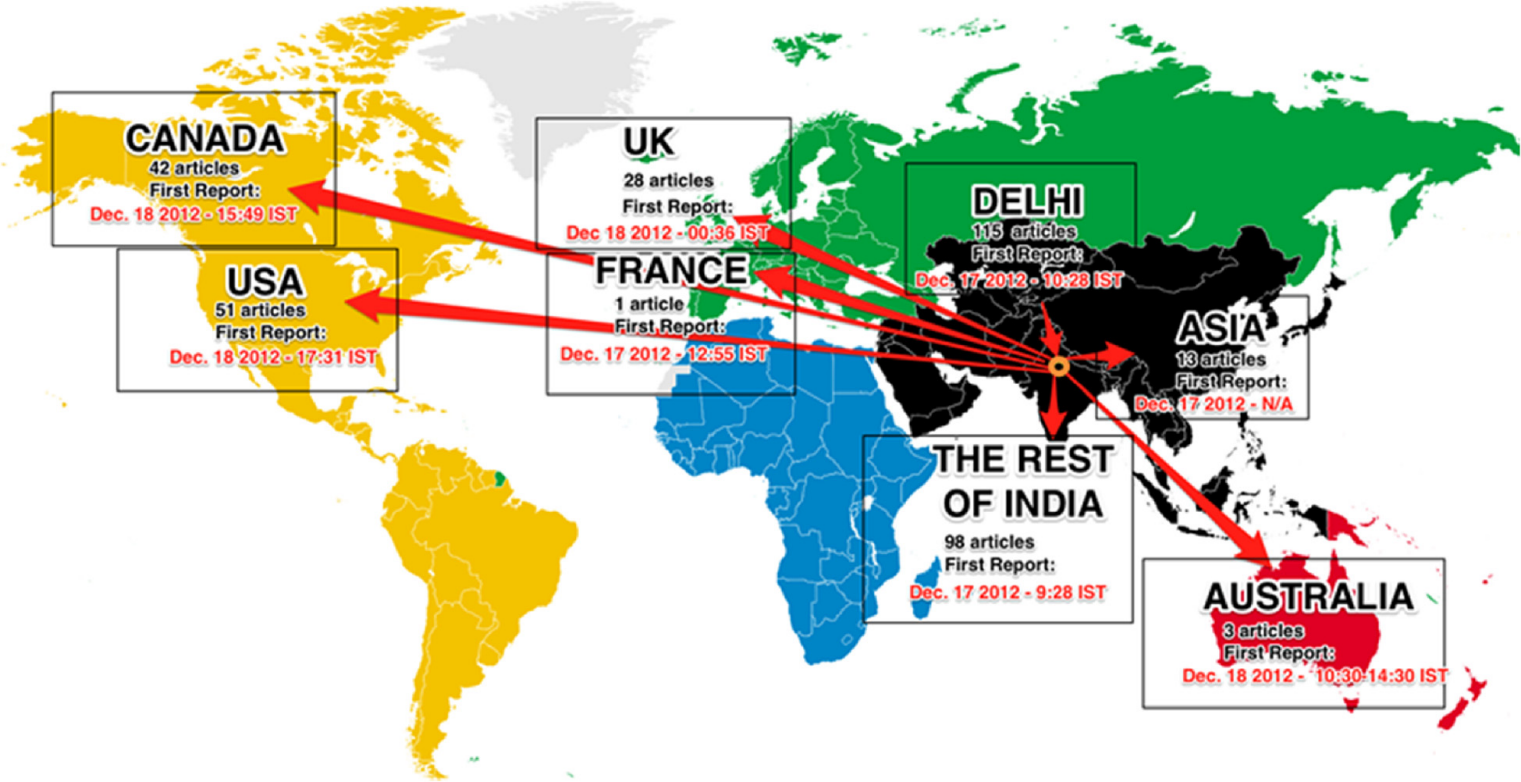
**Global distribution of media reports following events of December 16, 2012.**

**Figure 3:**
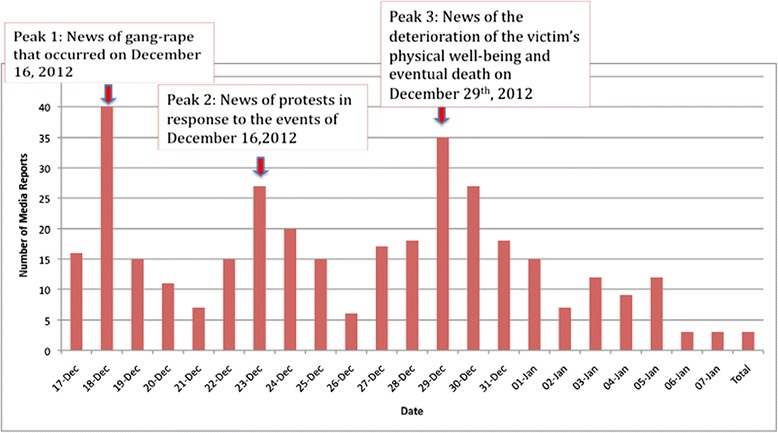
**Histogram on the number of reports published daily from December 16, 2012 to January 7, 2013.** N/A represents reports without a publication date.

**Figure 4:**
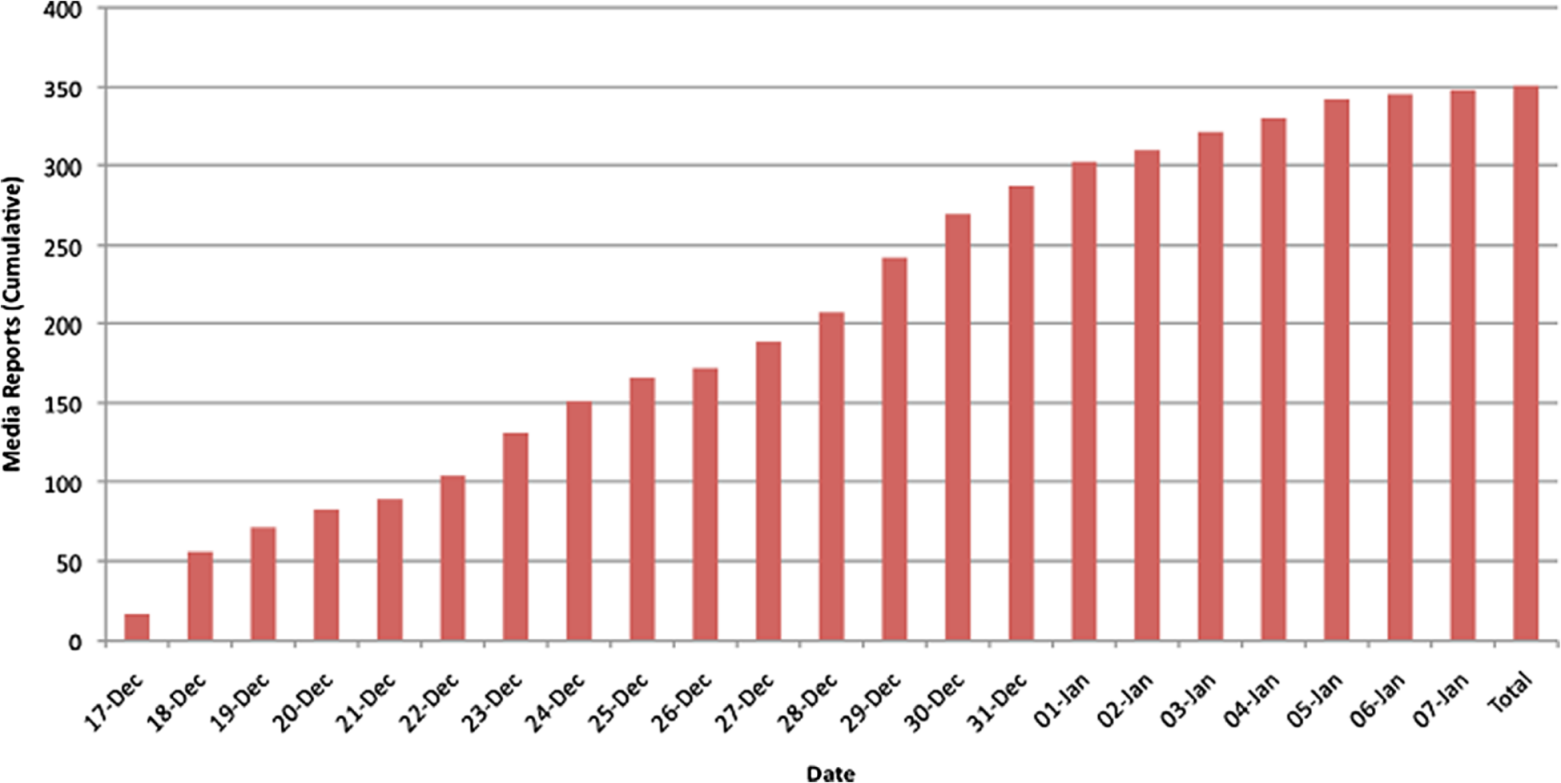
**Histogram on the number of cumulative reports published from December 16, 2012 to January 7, 2013.**

Reports in the media were most prevalent within India, and the following frequencies are the percentage of reports that originated in each region: Delhi (115/351, 32.8%), the rest of India (98/351, 27.9%), the United States (51/351, 14.5%), Canada (42/351, 12.0%), the United Kingdom (28/351, 8.0%), Asia (13/351, 3.7%), Australia (3/351, 0.8%), and France (1/351, 0.3%) (Figure 2). We identified the first report of the incident to be published by *The Hindu*, a national newspaper in India, on December 17, 2012 at 9:28 IST [20]. Following this incident report, local Delhi reports, reports in Asia, France, UK, Australia and then North American countries ensued. By December 18, 2012 the events had gained global recognition. Figure 3 depicts the number of reports published daily over the time from December 17th, 2012 to January 7th, 2013. Three spikes in publication occurred. The first spike occurred from December 17th, 2012 to December 19th, 2012 (71/351, 20.0%). The next spike of publications occurred from December 22nd, 2012 to December 25th, 2012 (77/351, 21.9%). The final spike of reports occurred from December 28th, 2012 to December 31st, 2013 (98/351, 27.9%). We identified a steady increase in reports and a plateau by the first week of January, 2013 (Figure 4).

### Incident reporting by media

Content analysis of 26 articles describing the events of the attack reported various details. Ten reports (44%) stated that the victim was assaulted by an iron rod, and her boyfriend was beaten. She was subsequently thrown from the bus (reported by 40%). Reports (20%) indicated the victims were robbed. The accused reportedly attempted to run over the victim (16%). Three reports state that the accused attempted to erase the evidence by washing the bus. From the three reports that indicate a time for the events, one report states that the victim was found at 9:15 pm on December 16 [21] while 2 reports indicate she boarded the bus at 9:45 pm [22,23]. The sources of evidence for these reports also varied. From the total, 20 articles provide sources for their evidence. Police statements or police reports are used by 90% of these articles, while 10% use claims from physicians to report on the victim’s condition.

There are 24 reports that showcase testimonies from the victim, her boyfriend and her family. Twelve reports discuss the boyfriend’s testimony and TV interview. In 66% of reports he describes that a bystander showed apathy when their bodies were discovered. He is quoted saying, "[passersby] slowed down, looked at our naked bodies and left," [23]. He also criticized the speed of response by the police (6/12, 50% of reports). Four reports discuss the family’s response. The father wishes to have his daughter named by the media to “inspire others” (3/4, 75%) [24]. All testimony articles provided evidence to support their claim, supplying quotes from the individual. The reports all provide one-sided views of the individual giving the testimony.

### Victim’s health condition reporting by media

There are 112 media reports that focused on the victim’s health or any medical decisions made pertaining to her health (theme 2). Of these reports, 52 (46.8%) described the event of the gang-rape. There were 23 reports (20.7%) that gave information on the victim’s transportation via helicopter to Singapore.

While 61 of the 111 reports (54.9%) stated that the victim was in serious condition, 37 reports (33.3%) discussed the multiple surgeries that had been performed in attempt to aid her. There are 43 reports (38.7%) that describe her condition as having internal bleeding and intestinal damage. Of the 111 reports, 13 of them (11.7%) stated that the victim had experienced brain damage. There are 4 reports (3.6%) that describe the victim’s brain damage as not being able to talk but being able to write down her thoughts. Three reports (2.7%) made reference to a statement made by the victim, “Ma main jeena chahti hoon”. (Translation: Mom, I want to live)” [5,25]. Within these 111 reports, there were 29 (26.1%) that released information that the victim had died and been cremated.

Analysis of the sources of data showed that 43/111 media reports provided specific evidence to support claims. The majority (25/43, 58.1%) quote the attending physician or a representative of the hospital, as their source of information. Six reports (13.8%) use quotes from visiting politicians to describe her condition. Three international (6.9%) papers use quotations from Indian newspapers to support their claims. Additionally, 4 reports (9.3%) used claims from family and friends. The remaining media reports (11.6%) use a combination of the above to support claims.

### Reporting of protests

There are 134 media reports identified which primarily discussed the protests as a result of the nationwide outrage. The earliest papers discussing the protest in Delhi were published December 18, 2012 [26]. Content analysis of reports showed that from the 134 reports, 74 reports (55.2%) indicate that protests were based on criticism of the current government and their lack of action in protecting women. Leader of the opposition, Sushma Swaraj is quoted asking, “What is the government doing to curb rape cases in the capital?” [27]. 28 reports (20.9%) emphasize that citizens were seeking justice for the victim and protesters demanded the death penalty. Additionally, 32 reports (23.9%) underscore both government criticism and the demand for justice as the underlying push for demonstrations.

Protests ranged from peaceful demonstrations to violent outbreaks. Media reports of violent protests (68/134, 50.7%) described police barricades at Jantar Mantar, a world heritage site in Jaipur and use of force to keep protestors controlled [28]. A total of 20 reports discuss the injuries and death of a constable as a result of the protests. All reports of violent protest occurred in and around Delhi.

Conversely, reports of peaceful protests (36/134, 26.7%) described candlelight marches and demonstrations. One report discussed how protestors appealed to people on social networking sites to gain support [29]. Although most papers discuss national outrage, eight reports (8/36, 22.2%) describe incidences of peaceful protests internationally. The first international report is of a silent protest in Toronto on January 3rd, 2013 [30].

Analysis of the sources of data showed that 107/134 media reports provided evidence for their information, while 27 did not. These reports were short (4–8 sentences) synopses of the event on a page containing other stories and/or lacked use of quotes and specific sources or photos to support the article arguments. From these 107 media reports, 66 (61.6%) provided quotes or photographs to demonstrate opinions of both protestors and government officials. Two media reports (2.2%) were photo galleries and only focused on the perspective of the protestors. Conversely other reports (39/107, 36.4%) only expressed the point of view of officials with regards to the protest.

### Opinions of victim reported by media

The 55 articles that address the general public’s response show a polarized representation. In 32 reports (58.1%) the public is characterized to have been supportive of the victim and shocked by the events. They applaud the victim’s bravery by giving her the symbolic name “Damini”, meaning Lightning, after the 1993 Hindi film with this name. The main character, Damini, is thought of as a hero who fights for equality and justice for a victim of rape [31]. In 23 reports (41.81%) the global outrage and anger surrounding the event is emphasized. The reports discuss the public’s demand for change in the treatment of women in India and concern for the safety of women in India [32]. The representation of citizens is not limited to India. Ten reports underscore international response and criticize the Indian government.

One report however discusses the controversial view of spiritual guru Asaram Bapu. He was criticized for stating, “The victim is as guilty as her rapists” [33]. Of the 55 articles addressing the public’s response, 6/55 (10.9%) described instances of victim blaming. One article provided a quotation from a Rajasthan state legislature stating that instances of rape would decrease if women wore pants instead of skirts [32]. These cases of victim blaming provided a general theme that women who do not dress conservatively cause rape to occur. Another theme present throughout these 6 articles is that women could reduce the occurrence of rape by not going out past sundown. These instances of victim-blaming provoked protest throughout the nation as people contested the idea that the women who are victimized hold responsibility in cases of rape.

The sources used by these articles, to support their claim, represent views of individual citizens, including a large number of students (80.7%), government or police officials (14.3%), and photographs (5.3%).

## Discussion

In India, there were 24,923 rape cases reported to the Ministry of Home Affairs in 2012, representing an increase of 15.8% from 2009 [34]. It is important to consider that rape is a crime that is vastly under-reported and consequently, estimates of incidence frequently under-estimate the true nature of the problem, including in India [35]. There has been a history of protest in India regarding the topic of violence against women, and many people in India have fought to gain gender equality as well as tougher punishment for rapists. Recently, protests have spread throughout India and the rest of the world after a 23 year-old student was brutally gang-raped on a moving bus in South Delhi in December 2012. The protests fight to have even harsher punishment for rapists, including the possibility of the death penalty [10]. Professional media plays a major role in spreading the information of an event such as this rape, as well as information on the protests and public response to the event. This specific rape has sparked a major response within the media on a global scale. This major response is critical in the development of awareness regarding women’s health issues, and the facilitation of the media to create this response should be utilized in future events pertaining to violence against women and other women’s health issues.

The results of this study show that the news of the gang-rape in Delhi on December 16, 2012 spread globally through professional media sources within two days. This transition followed a pattern in which Indian news sources provided the first reports and international reports were delayed by a day. By December 18, 2012 the news of events in Delhi was globally distributed. Interestingly, the first English language report identified by our study was not made by a local Delhi paper. *The Hindu*, which published the first report, is a prominent national newspaper.

The results also indicated three peaks in the dispersion of media reports during major events in the timeline, before a final plateau of reports is established. The first correlates to a large number of reports published regarding the occurrence of the event on December 16th, 2012. The second peak of media reports occurred in response to the formation of protests over the incident from December 22nd, 2012 to December 25th, 2012. The last peak from December 28th, 2012 to December 31st corresponds to the deterioration of the victim’s physical well-being and eventual death on December 29th, 2012. Overall, the number of reports increased steadily during the period of December 17th to January 7th, while beginning to decline near the end. The plateau forms as information of the perpetrator’s custody begins to overtake the media’s attention. The media shifts from emphasis on the protests and begins to focus on the trials of the accused men. The relatively short delay in initial reports of the event to the emergence of protests suggests the efficiency in which the spread of information by media is capable of creating social movements.

It is valuable to know how media spreads after an event similar to this because it demonstrates how effective and fast the media is as a means to disseminate information. This study is specifically focused on the issue of violence against women, but the informative nature of media and ability to spark social movement can be applied to women’s health issues in general that require global attention. Considering the impact of the media as demonstrated by this study, those interested in creating awareness among the general population need to recognize the importance of engaging the media in pursuing their desired outcomes [15]. Although the media is a quick source of creating awareness, it also has limitations. One such limitation assessed in our study is the variability of information provided. Media reports were highly variable in content, even if the focus was on the same theme. Additionally, analysis of their sources of evidence showed high heterogeneity among the reports. Although media sources are not entirely heterogeneous in content, they have demonstrated a pivotal role in social movement development in this specific case, which can be valuable in future cases of social movements surrounding women’s health issues.

### Strengths and limitations of this study

There are three main strengths within this study: 1) Each included news source was read in full before being nominated for inclusion, instead of assessing solely based on the title; 2) large and comprehensive databases for published news were used when searching for articles, including Factivia and LexisNexis media databases; 3) The screening for selection of media analysis and author bias analysis was carried out independently in duplicate, preventing expectation bias from a single rater process and increasing reliability.

The six main limitations within the study are: 1) The study was solely focused on online media sources, which may prevent small, local media sources from being included in the results. Written newspapers and televised local news would be sources of media that are not present in the results due to this limitation; 2) Some online sources may have been deleted or archived due to the elapsed time in between the event and when the study had been performed; 3) The poor inter-rater agreement indicates that there was high variability in the methods of assessment between each rater. This could be the result of poorly defined criteria to assess author bias. There are no tested tools to assess bias in media sources systematically, which we could have used. Additionally, it may be argued that all media sources are subjective, thus making a distinction is difficult. In future studies, assessment methods pertaining to the assessment of author bias within a media source should be clearly defined in order to increase the inter-rater agreement; 4) Social media was not considered in this article. The spread of information of the event on a global scale would have also been affected by social media. 5) Manual searching of common news sources in English was conducted, which eliminates a large number of potentially relevant articles that are not in English. The timeline may be more accurate if sources in other languages were also included. 6) Attempts were made to find exact times for all resources, however in the case of reports for Indo-Asian countries a specific time could not be identified for the first report from this area. This is a limitation of news sources that will affect most media analyses.

### Future directions

This study provides assessment of the timeline corresponding to information translation through media sources after an event of interpersonal violence such as the New Delhi gang-rape on December 16, 2012. It also provides insight into what details are presented by the media including: bias towards the event, included and excluded facts. Future studies may look further into the idea that media spreads very quickly after a major event like this; however the information that is spread is highly variable in its contents. Assessing the accuracy of a large group of media reports pertaining to the same topic will aid in determining the factual accuracy and details found within media reports that are released quickly after a major event.

Secondly, this study focused only on professional journalism. Future research can look at how information transitions through social media sources and citizen journalism. This study would provide a more complete image of information translation in the mass public.

## Conclusion

The gang-rape in Delhi on December 16, 2012 provides a model for the spread of information across the globe through the media. The timeline of this spread shows that in a short period of time, information is able to reach across the globe through major media sources. This spread is a key contributor to the development of social movements pertaining to violence against women, as demonstrated by the case studied in this project. The details of the event that are found within these articles are variable, resulting in many different variations of the story. These findings suggest that although online media facilitates a rapid spread of information, there are real risks of inaccurate initial reporting that are often perpetuated by subsequent reports. Ultimately, the rapid spread of information by the media may be variable, yet it acts as a powerful tool in the formation of social movements around acts of violence against women such as this case.
